# Is the Cultural Transmission of Irrelevant Tool Actions in Adult Humans (*Homo Sapiens*) Best Explained as the Result of an Evolved Conformist Bias?

**DOI:** 10.1371/journal.pone.0050863

**Published:** 2012-12-12

**Authors:** Nicola McGuigan, Daryl Gladstone, Lisa Cook

**Affiliations:** School of Life Sciences, Heriot Watt University, Edinburgh, United Kingdom; Université de Strasbourg, France

## Abstract

**Background:**

Recent studies of social learning have revealed that adult humans are “over-imitators” who frequently reproduce a model's causally irrelevant tool actions to the detriment of task efficiency. At present our knowledge of adult over-imitation is limited to the fact that adults do over-imitate, we know very little about the causes of this behavior. The current study aimed to provide novel insights into adult over-imitation by extending a paradigm recently used with human children to explore social aspects of over-imitation. In the child study observers saw two models demonstrate a tool-use task using the same inefficient approach, or two models demonstrate different approaches to the task (one inefficient and one efficient). The manipulation of social influence came in the testing phase where the observer completed the task in the presence of either an inefficient model or an efficient model.

**Methodology/Principal Findings:**

We adapted the paradigm used in the child study to provide the first systematic exploration of factors which may lead to adult over-imitation including: 1) the presence of the model(s) during testing, 2) the presence of a competing efficient task demonstration, 3) the presence of a majority displaying the inefficient approach, and 4) the ‘removal’ of the experimental context during task completion. We show that the adult participants only over-imitated in conditions where the inefficient strategy was the majority approach witnessed. This tendency towards over-imitation was almost entirely eliminated when the participants interacted with the task when they believed the experiment to be complete.

**Conclusions:**

Our results suggest that adult over-imitation is best explained as a result of an evolved ‘conformist bias’ argued to be crucial to the transmission of human cultural behavior and one which may be unique in the animal kingdom.

## Introduction

Within the social learning literature a somewhat curious phenomenon has recently received a great deal of attention. The phenomenon in question has been dubbed ‘over-imitation’ [1], or ‘over-copying’ [2], to describe instances where observers not only copy the actions performed by a model they do so to such an extent that task efficiency is actually reduced (for example by copying causally irrelevant elements of an action sequence). The study of over-imitation has almost exclusively been confined to early human development where a plethora of studies have revealed a consistent pattern of over-imitation across tasks, contexts and cultures [3]–[8]. Intriguingly this propensity towards high fidelity imitation has been found to be lacking in our closest primate cousins *Pan troglodytes* [3] and *Pongo pygmaeus* [9]. A further intriguing finding from the over-imitation literature is that within the human species (*Homo sapiens*) over-imitation increases with age [10], with adults performing causally irrelevant tool actions with higher levels of fidelity than preschool children [11], [12]. However, the finding that adults are more over-imitative than children has been somewhat overlooked in the literature, a neglect which is rather surprising as there would appear to be good developmental reasons why children should faithfully copy the actions (even those that appear functionless) that they witness, but why adults would do so is less clear. The aim of the current study was to fill this gap in our knowledge by systematically varying elements of the social context (e.g., the number of individuals demonstrating the inefficient strategy) in order to explore the impact on the propensity of adults to over-imitate in a tool-use task.

The occurrence of over-imitation appears at first glance surprising, as it suggests that rather than selecting to copy relevant and useful information observers are blindly copying all elements of the display, relevant or otherwise. This somewhat ‘blind’ copying would appear to degrade the usefulness of imitation as a quick and effective strategy for learning from other individuals within our experimental context. The challenge for researchers interested in over-imitation is to explain why such an adaptive strategy as imitation should on occasion spill over into inefficiency? A suite of theories have been proposed in order to explain the occurrence of over-imitation in childhood, with the most prominent stemming from social and cognitive perspectives. There are many aspects of the social environment proposed to lead to over-imitation in childhood including a desire to share experience with, affiliate with, or be ‘like’, the model, or to meet the perceived expectations of the model [13], [14]. A more recent social theory has proposed that children may be over-imitating due to the acquisition of a prescriptive norm of what ought to be done with a particular object [4], [5]. In contrast cognitive theories of over-imitation have proposed that the actions performed by a model are viewed as intentional and either automatically copied due to a distortion in causal beliefs [1], [15] or through viewing the model's irrelevant actions as having a purpose (even though that purpose may be unclear to the observer) [3], [7].

Although theories of over-imitation are plentiful few studies have attempted to tease apart the theories directly. One recent attempt to disentangle the social and cognitive explanations of over-imitation was conducted by Nielsen and Blank [14]. In this study 4- and 5-year old observers (the term observer refers to the test participant throughout) were allowed to witness two adult models retrieve a reward from inside a box using one of two strategies. One strategy was to retrieve the reward from the box using only causally relevant tool actions (efficient approach), whereas the alternative strategy was to retrieve the reward after performing a series of causally irrelevant tool actions (inefficient approach). In a modeling phase the children witnessed task demonstration by either two inefficient models or one inefficient model and one efficient model. The crucial manipulation came in the testing phase where one model left the testing room leaving the child to perform the task in the presence of either the efficient model or an inefficient model. The authors predicted that if over-imitation is the result of attempting to be like the model then the participants should copy the approach of the person who remained with them during testing, whereas the theory of automatic copying would predict over-imitation across conditions as each participant would have observed at least one demonstration containing the irrelevant actions. The results appeared to provide some support for the social theory as the children only over-imitated in the conditions where the inefficient model remained in the testing room, and acted more selectively when only the efficient model remained.

However, the causes of adult over-imitation remain relatively unexplored. Are adults as influenced by the social context as children? In order to explore this possibility the current study aimed to replicate and extend the paradigm used by Nielsen and Blank with a group of adult participants. More specifically Experiment 1 aimed to replicate the three conditions presented by Nielsen and Blank (i.e., one model always remained in the testing room) with one crucial procedural addition. In Nielsen and Blank the model who remained in the testing room always presented the box to the participant leaving open the possibility that the participants were simply adopting the rule ‘copy the approach of the model who hands me the box’. We aimed to control for this possibility in the current study by systematically varying which of the two models presented the box to the participant. It was predicted that the adult participants would over-imitate after observing two inefficient models, however it was less clear how the participants would perform in the mixed strategy conditions. It may be the case that the adults would be influenced by the presence of the model and adopt the approach of the model who remained in the testing room, alternatively simply viewing an efficient approach may lead the participants to perform the task more efficiently. A further aim of Experiment 1 was to explore the impact of model presence in more detail by presenting additional novel conditions where both models were either present (maximum social influence) or absent (minimum social influence) from the testing room whilst the participants completed the task. If the presence of the models influenced task performance then we would predict that the highest levels of over-imitation would occur when both models remained in the testing room and the lowest levels when the participants attempted the task by themselves. A second experiment aimed to provide the first detailed exploration of the role of conformity to the majority strategy in over-imitation. This was achieved by: 1) increasing the number of inefficient strategies modeled relative to the number of efficient strategies (3∶1) and 2) by eliminating the pressure to conform by presenting the participants with the task outside of the experimental context. Recent studies with young children have shown that individuals are influenced by their peer group early in development [16], [17], [18]. It was therefore predicted that if the participants would adopt the majority approach witnessed and would subsequently be more likely to over-imitate when presented with a 3∶1 (inefficient: efficient) strategy ratio than they would when presented with an equal ratio of each strategy, and would be less likely to over-imitate when completing the task outside of the experimental context.

## Experiment 1: Procedure, Results, and Discussion

**Figure 1:**
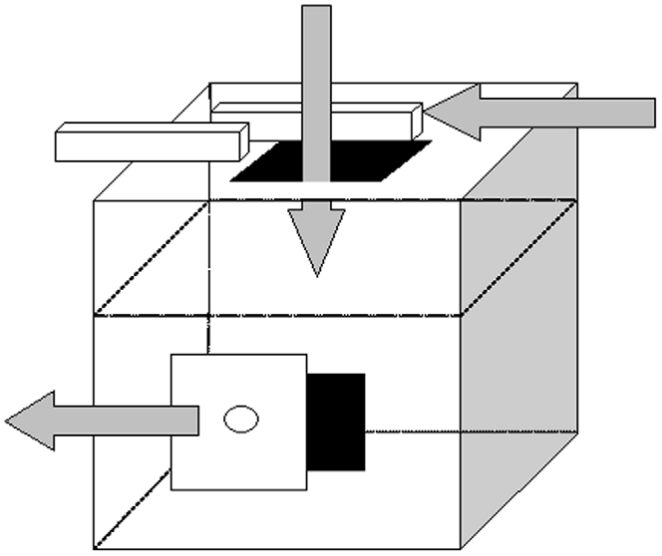
Illustration of principal task components. (1) Remove bolts. (2) Irrelevant tool insertion, tool strikes barrier within box 3 times. (3) Open door in order to retrieve the reward. The inefficient technique included all 3 task components whereas the efficient technique comprised component 3 only.

We began by presenting a group of adult participants (n = 84, 37 males, mean age 21 years, range 17 to 53 years) with two task demonstrations in which they viewed two adult models (both female) use a tool to retrieve a reward from inside a puzzle box (Figure 1.) The reward was either retrieved from the box using only causally relevant actions (efficient approach), or was retrieved after the model had performed five causally irrelevant actions (inefficient approach). Thus inefficiency in the current task is recognized by the reproduction of superfluous actions, a measure of inefficiency which has been shown to relate directly to time taken to complete the task (i.e., the more superfluous actions performed the longer the task takes to complete) [6]. The participants observed either two inefficient models (inefficient strategy condition), or one efficient model and one inefficient model (mixed strategy condition), before being allowed to attempt the task in the presence of one, neither or both models.

### Influence of Model Presence: Inefficient Strategy Conditions

**Figure 2:**
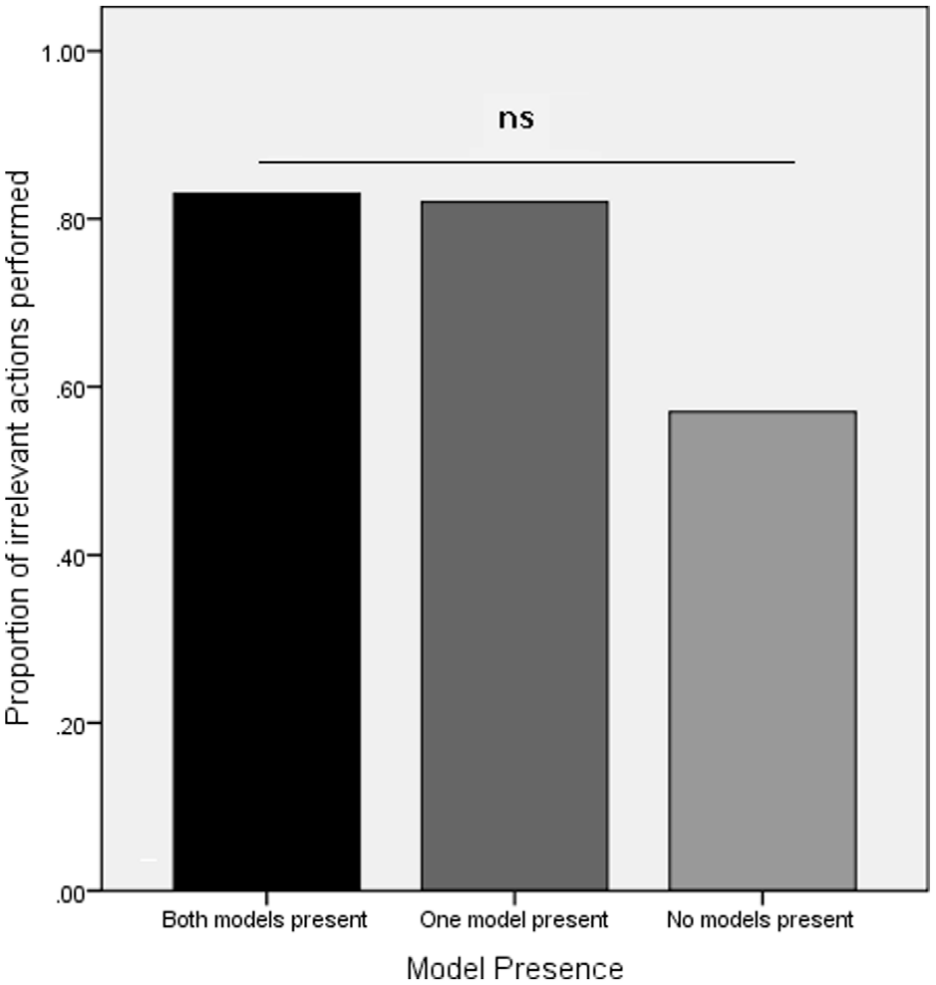
Proportion of irrelevant actions performed in the inefficient strategy conditions of Experiment 1 as a function of model presence.

In order to explore the impact of model presence on over-imitation the total number of irrelevant actions (max = 5, min = 0) performed by the participants in the three inefficient strategy conditions were analyzed using a univariate ANOVA with model presence (one, two or no models present) as a between-participants factor. If model presence was impacting on performance we would expect to see high levels of over-imitation in the two conditions where there was at least one model present and a substantially reduced level of over-imitation in the both models leave condition. However, the analysis revealed no significant effect of model presence (Figure 2; F (2, 33) = 2.3, p = .11, μ = .12) with the participants in the each of the inefficient strategy conditions performing a large number of irrelevant actions (mean irrelevant actions: one model stays = 4.1; both models stay = 4.2; both models leave = 2.8) irrespective of the presence of the model(s) during testing.

### Influence of Model Presence: Efficient Strategy Conditions

**Figure 3:**
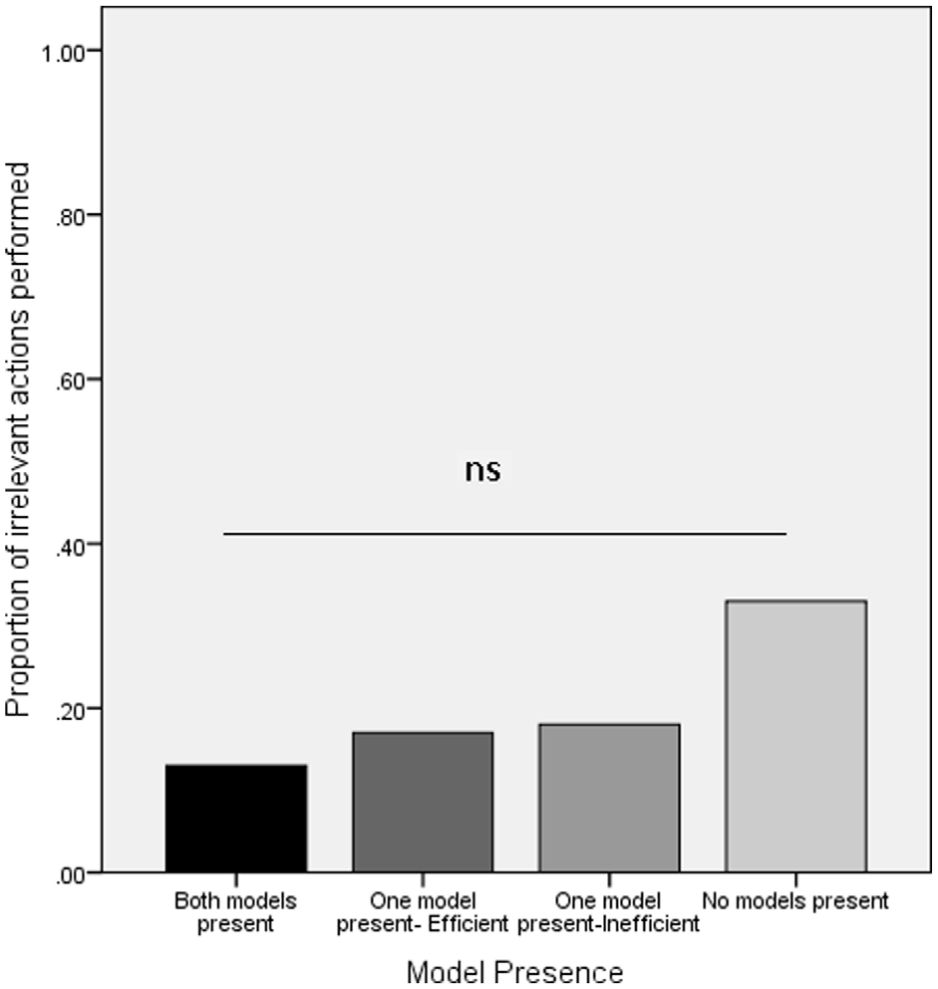
Proportion of irrelevant actions performed in the mixed strategy conditions of Experiment 1 as a function of model presence.

As well as exploring the impact of model presence in the inefficient strategy conditions we were also interested in how the presence of the model(s) would impact on performance in the mixed strategy conditions. If the presence of the model(s) was playing a key role in determining the strategy adopted by the observer we would expect little over-imitation in the both models leave condition, a mixed response in the one model stays condition (depending on which particular model remained), and an intermediate level of over-imitation in the both models stay condition. As with the inefficient strategy conditions the total number of irrelevant actions performed was analyzed using a univariate ANOVA with model presence (one (inefficient or efficient), two or no models present) as a between-participants factor. The analysis revealed no significant effect of model presence (Figure 3; F (3, 44) = .91, p = .44, μ = .06) with the participants in the each of the mixed strategy conditions performing very few irrelevant actions (mean irrelevant actions: efficient model stays = 0.8; inefficient model stays = 0.9; both models stay = 0.7; both models leave = 1.7) irrespective of whether the model(s) were present or absent during testing.

These results show that the observers performed most over-imitation in the three conditions where they were exposed to two inefficient models (see Table S1 for detailed condition breakdown). This occurred irrespective of whether there was one model, two models, or no models present during testing, suggesting that the observers were not over-imitating as a result of the physical presence of the model(s)in the testing room. The participants in the mixed strategy conditions also appeared uninfluenced by the presence of the model(s), with the majority of participants (65%) opting to perform the task using the efficient approach (as compared to only 11% in the both models inefficient condition). The adoption of the efficient approach occurred even in the presence of the inefficient model suggesting that, unlike the children in Nielsen and Blank, the adult participants were not adopting the approach of the model who remained in the testing room. However, there are at least two possible explanations for the reduced levels of over-imitation witnessed in the mixed strategy conditions. It is possible that the presence of an alternative, more efficient technique, alerted the participants to the possibly that the reward could be obtained more readily simply by inserting the tool in the lower hole. Second, it may be that the participants performed less efficiently in the inefficient strategy condition as they were conforming to the majority approach witnessed (2 inefficient vs. 0 efficient strategies). This majority strategy contrasted with the position of the observers in the mixed strategy conditions who saw the inefficient strategy in a 1∶1 ratio with the efficient strategy. Perhaps with an equal strategy ratio the participants opted for the more efficient approach.

The aim of Experiment 2 was to tease apart these explanations by presenting two novel conditions in which the observer witnessed the efficient strategy and the inefficient strategy in varying amounts (25% versus 75% of demonstrations respectively). This was achieved in two ways, first by increasing the number of models demonstrating the inefficient approach relative to those demonstrating the efficient approach (model majority condition), and second by having one efficient model and one inefficient model as in Experiment 1 but increasing the number of times the inefficient model demonstrated the task (strategy majority condition). If the presence of an efficient demonstration alone was enough to prevent over-imitation then we would predict that very few irrelevant actions would be witnessed in either condition as one model always demonstrated the efficient approach. If however over-imitation was the result of the inefficient approach being the most frequent strategy observed then we would predict that over-imitation would occur in both the model majority and the strategy majority conditions as each have a 3∶1 strategy ratio. Alternatively, it may be that participants would only over-imitate if they witnessed the majority of a group adopt this approach. If this is the case then we would predict that over-imitation would occur in the model majority condition only.

## Experiment 2A: Procedure, Results, and Discussion

We presented a new group of undergraduate students (n = 31, 10 males, mean age 20 years, range 18 to 36 years) with four task demonstrations, three demonstrations where the reward was retrieved from the box using the inefficient strategy and one demonstration where the reward was retrieved using the efficient strategy. Sixteen of the participants (majority strategy condition) observed two models (both female) perform all four of the demonstrations (one model performed three inefficient demonstrations, and one model performed one efficient demonstration), whereas the remaining fifteen participants (majority model condition) observed four different (all female) models (three inefficient models and one efficient model) perform the task.

### Model Majority versus Strategy Majority

**Figure 4:**
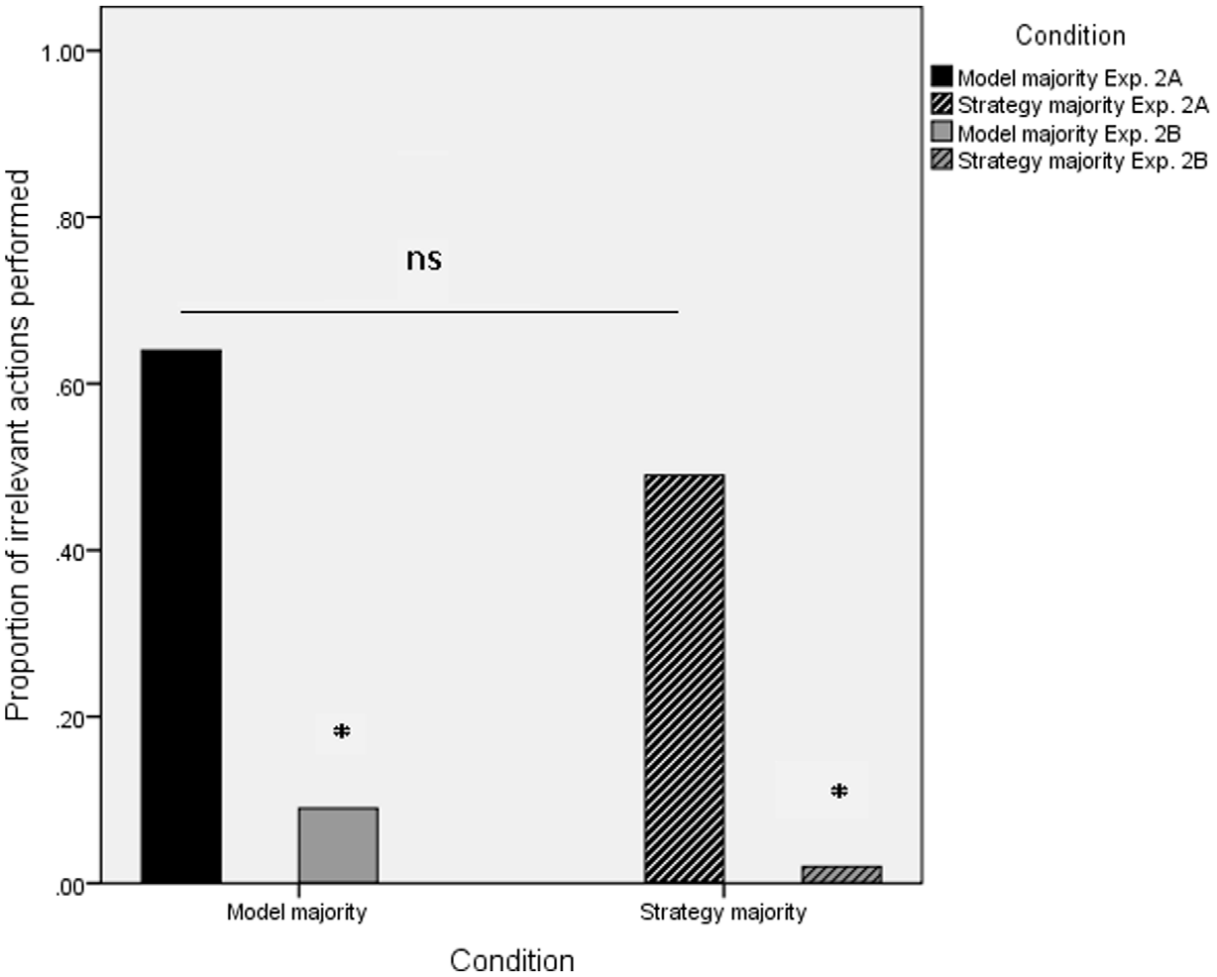
Proportion of irrelevant actions performed with box 1 in the model majority and strategy majority conditions of Experiment 2A and with box 2 of Experiment 2B.

Of interest was whether the number of irrelevant actions performed by the participants would vary according to whether they witnessed four models or two models perform the four task demonstrations. In order to explore the impact of model versus strategy frequency the total number of irrelevant actions performed by the participants in Experiment 2A were subjected to a univariate ANOVA with condition (model majority or strategy majority) as a between participants factor. The analyses revealed no significant difference in the number of irrelevant actions performed between the conditions (Figure 4; F (1, 29) = .92, p = .35, μ = .03) with participants copying many of the irrelevant actions in both the model majority condition (mean irrelevant actions = 3.2) and the strategy majority condition (mean irrelevant actions = 2.4).

### Factors which Influence Over-imitation (Experiments 1 and 2A)

Across Experiments 1 and 2A it appeared as though there were two factors which may have influenced the levels of over-imitation witnessed: 1) the presence of an efficient task demonstration, and/or 2) the presence of a majority inefficient strategy (either 2∶0 or 3∶1). In order to explore the impact of each of these factors we collapsed the data from the nine conditions of Experiments 1 and 2A into three groups. Group 1 (2∶0 ratio) comprised the three conditions were no efficient strategy was modeled (inefficient strategy conditions of Experiment 1). Group two (1∶1 ratio) consisted of the four conditions where the inefficient and efficient strategies were presented in equal frequencies (mixed strategy conditions of Experiment 1). The final group (3∶1 ratio) was made up of the two conditions of Experiment 2A where there was an increased ratio of inefficient strategies to efficient strategies. In order to explore whether there were differences between the three groups the total number of irrelevant actions performed by the participants was subjected to a univaiate ANOVA with group (2∶0 ratio, 1∶1 ratio or 3∶1 ratio) as a between-participants factor. The analysis revealed a significant effect of group (F(2, 112) = 23.3, p<.001, μ = .29), with Post hoc analyses (LSD tests with Bonferroni correction applied, adapted significance level .0167 for 3 comparisons) revealing that the participants in the 1∶1 ratio group (mean irrelevant actions = 1.0), performed significantly fewer irrelevant actions than those in the 2∶0 ratio group (mean irrelevant actions = 3.7, p<.001), and the 3∶1 ratio group (mean irrelevant actions = 2.8, p<.001). This suggests that the presence of an inefficient strategy majority was having a large influence on the occurrence of over-imitation, whereas the presence of an efficient strategy only prevented the occurrence of over-imitation when presented in a 1∶1 ratio with the inefficient strategy. The difference between the 2∶0 and 3∶1 groups did not reach significance. A full breakdown of condition comparisons is provided in SI Table 2.

## Experiment 2B: Procedure, Results, and Discussion

The results from Experiments 1 and 2A suggest that the presentation of an unequal ratio of inefficient to efficient task demonstrations had a substantial impact on the likelihood that the participants would over-imitate. These high levels of over-imitation may have resulted from the participants adopting the most common approach witnessed, in other words conforming to the majority strategy. In order to further explore the influence of conformity Experiment 2B aimed to present the task outside the context of the experiment, thereby removing the pressure to conform and allowing the participants to potentially display their real knowledge of the task (i.e., perform only the causally necessary actions).

The same participants who took part in Experiment 2A were tested in Experiment 2B. On completion of Experiment 2A one of the experimenters pointed to a second box (located on a shelf directly behind the participant, out of reach of the experimenters) and said to the participant ‘*that they (the experimenters) were going to use another box for the next participant, could you (the participant) please check to see if the reward is inside the box?*’ In order to explore whether the participants in Experiment 2B would perform the irrelevant actions outside of the experimental context we compared the number of irrelevant actions performed with box 1 (Experiment 2A), to those performed on the ‘post experiment’ box 2. A repeated measures ANOVA revealed that the participants performed significantly fewer irrelevant actions with the ‘post experiment box’ in both the model majority condition (Figure 4; mean irrelevant actions box 1 = 3.2, box 2 = 0.5; F(1, 14 = 28.6, p<.001, μ = .67), and the strategy majority condition (mean irrelevant actions box 1 = 2.4, box 2 = 0.1; F(1, 15 = 15.8, p = .001, μ = .51), suggesting that the participants responses were heavily influenced by the experimental context.

## Discussion

Taken together the results of Experiments 1 and 2 demonstrated that adult humans: 1) consistently over-imitated in conditions where the inefficient technique was in the majority, 2) always acted efficiently in the 1∶1 conditions irrespective of the presence of the model(s) and 3) showed no evidence of over-imitation outside of the experimental context. These findings paint a picture of adult humans as adopting the majority behavior witnessed but not necessarily adopting the technique of the particular model present. This pattern of performance shares some similarities and differences to that of the children from previous studies [1], [3], [14]. Like adults children faced with a majority inefficient strategy frequently over-imitated irrespective of whether the model(s) were present or absent from the testing room [3]. However, unlike adults, when presented with two alternative strategies children tended to adopt the approach of the model present, irrespective of strategy efficiency [14]. A difference between adults and children was also evident outside of the experimental context where Lyons et al. (2007) found that, unlike our current adults, children frequently continued to over-imitate when the experiment was ‘complete’ [1].

These discrepancies in performance suggest that over-imitation may stem from a variety of mechanisms that differ from observer to observer. With respect to over-imitation in childhood the finding that some children continued to over-imitate outside of the experimental context suggests that there may be some distortion in cognitive processing not evident in the current adult sample, for example through continuing to attribute an unknown purpose to the model's intentional actions [3], [7]. However, as well as cognitive influences children also appear to be heavily influenced by their social environment as evidenced by the selective copying witnessed in Nielsen and Blank. This affiliative tendency may be particularly strong in the case of young children who are viewing models much older, and subsequently more dominant, than themselves [11]. There is also evidence to suggest, through verbal exclamations, that some young children over-imitate due to the acquisition of a prescriptive norm of how the object works [4], [5].

It therefore appears that the routes to over-imitation, particularly in childhood, may be varied and comprise both social and cognitive mechanisms. However, the performance of the current adult participants suggests that they were predominantly influenced by their social world, continually adopting the majority approach witnessed. The tendency of our adults to veer towards the majority approach suggests that they were best described as conformists, perhaps even “over-conformists” [19]. Whiten proposed that over-conformity occurs in instances where actions are copied despite the presence of counter evidence, resulting in the copying behavior being somewhat maladaptive. This tendency towards over-conformity was particularly striking in the instances where the participants witnessed, and subsequently ignored, an efficient technique in favour of an inefficient task variant. The inefficiency of the participants in the experimental context contrasted sharply with the almost complete lack of irrelevant actions performed when the participants believed that the experiment was over. This increased efficiency clearly demonstrated that the participants understood the causality of the task, so why were the participants so conformist within the experimental context?

One reason that the participants may have evidenced over-conformity is that in such a thoroughly cultural species such as ourselves it is adaptive to copy the approach of the majority of those around you. Richerson and Boyd [20] proposed that evolution has equipped humans with a set of “fast and frugal” (p. 119) heuristics which allow individuals to select the optimum behavior without expending energy trying out various alternatives. One such heuristic is a ‘conformist bias’ where in general it pays individuals to copy the most common behavior witnessed as this behavior has likely been selected as the most adaptive through processes such as natural selection, guided variation and content bias [20]. Clearly a heuristic such as this would be extremely beneficial in the majority of circumstances, however it also has the potential to allow an individual to inadvertently copy maladaptive traits. In this sense the over-imitation witnessed is not ‘foolish’ or ‘silly’ rather it is the result of an over-extension of a powerful social cognition that is the bedrock of human culture [2], [19].

As well as providing the first empirical evidence of conformity in adult over-imitation the current results may also shed some light on the type of conformity that is taking place. This question has been somewhat neglected outside of the social psychology literature, and is one which has recently begun to stimulate a great deal of interest in animal social learning [21]. Cialdini and others [22]–[25] have proposed that conformity can result from an individual attempting to behave accurately in a situation where psychological uncertainty is high (informational conformity), or through an individual attempting to manage social relationships (normative conformity), suggesting an affiliative motive to conformity. Similarly, with more direct reference to over-imitation, Kenward et al. [4], [5] have suggested that instances of over-imitation may stem from the adoption of a prescriptive norm. In support of the norm theory there are various features [21] of the current participants' performance which suggest that normative conformity was prevalent: 1) the behavior stopped outside of the experimental context, 2) the individual acted on unreliable social information, and 3) the participants displayed the most frequent behavior witnessed even when it was not optimal [20]. This pattern of behavior suggests that these individuals were concerned with social aspects of the environment, perhaps attempting (not necessarily consciously) [26] to portray a positive image of themselves or maintain positive social interactions with the models [21], [23]. Intriguingly normative conformity was witnessed regardless of how the inefficient majority was achieved (i.e., either through a model majority or a strategy majority), information which appears counter to our intuitive understanding of the results of the seminal social psychology studies of Asch [27], [28], and provides crucial information for the mathematical modelling of cultural transmission [29], [30]. The current results also suggest that the removal of the models from the testing room may not have prevented conformity from occurring. The participants in the both models inefficient condition of Experiment 1 continued to over-imitate when both models left the testing room suggesting that the adult participants may have continued to conform, perhaps deeming themselves to still be under experimenter observation.

In contrast to the high levels of over-imitation witnessed in the inefficient majority conditions 65% of the adult observers in the 1∶1 ratio conditions were able to over-ride social information in order to perform the task efficiently (see SI Table 3 for a detailed breakdown of performance). This suggests that the presence of one inefficient task demonstration was not sufficient to induce normative conformity when a more efficient counter strategy was witnessed. However, it may be that we are witnessing informational conformity in these conditions. If we view the observer as part of the group [31] then the strategy balance switches from a 1∶1 ratio to a majority efficient approach (2 efficient versus 1 inefficient), perhaps leading to engagement in informational conformity. Indeed, many characteristics of informational conformity were evident [21] in the mixed strategy conditions of Experiment 1 including: 1) the observers displayed the more frequent optimal behaviour, 2) the observers relied on their own experience rather than unreliable social information, and 3) the efficient strategy was adopted outside of the experimental context. However it appears as though informational conformity was limited to instances where the task variants were presented in a 1∶1 ratio, or when the conflict between the variants was removed by leading the participant to believe the experiment was complete. In every other instance the participants appeared to be engaging in normative conformity, thereby neglecting their own causal knowledge, and engaging in a ‘foolish’ strategy which may be the most powerful cultural tool available to *Homo sapiens*.

## Materials and Methods

### Ethics Statement

The study was approved by the Life Sciences Ethics Committee at Heriot Watt University (Application 2011:113) and all participants provided written informed consent.

### Apparatus

The task used in the current study was a tool based task which has been successfully employed with adults in previous studies [11], [12]. The end result of the task was to withdraw a reward (a magnet backed slip of paper saying ‘Congratulations!’) from inside a transparent puzzle box using a magnet tipped probe. The reward was withdrawn by the model using one of two strategies each of which differed in the level of efficiency. The efficient action sequence required the model to use only causally relevant actions to retrieve the reward (open a small door on front of the box and insert the probe into the hole behind). In contrast the inefficient action sequence required the model to perform five causally irrelevant actions (uncover a hole on top of the box by removing two bolts, insert the probe into the top hole and strike a false ceiling inside the box three times), before successfully using the probe to withdraw the reward from the lower hole. Of interest was whether participants would copy all of the actions, irrespective of their causal relevance (i.e., over-imitate), or perform only the actions necessary to obtain the goal. Two different, but almost identical, versions of the box were used. In Experiments 1 and 2A the box used was constructed from thin Perspex for use in human studies whereas the box used in Experiment 2B was identical in every respect with the exception that it was constructed from thicker Perspex for use in comparative studies.

### Procedure

In Experiment 1 the participants observed two adult female models perform either two inefficient task demonstrations or one inefficient and one efficient task demonstration before being allowed to interact with the task in the presence of one model, both models or neither of the models. In Experiment 2A the ratio of the inefficient to efficient strategies was increased by increasing the number of models demonstrating the task or by increasing the number of times an individual model demonstrated the inefficient strategy. Across experiments the instructions given to the participants during task demonstration, *“watch this”*, and task presentation, *“now it's your turn”*, were minimal. In the one or both models leave conditions of Experiment 1 the circumstance in which the model(s) left the testing room was designed to be as natural as possible. In the one model leaves conditions the departing model said: *“I'm sorry that's my phone, I've got to take this”* before leaving the testing room. The script was similar in the both models leave conditions with one model saying “*I'm sorry that's my phone I've got to take this*” before going on to say to the other model, *“actually, that's X (a mutual friend) she will want to speak to you too”* before both left the testing room. The models encouraged the participant to interact with the box in their absence *“we will just be a minute, you continue”* before leaving the testing room. The models surreptitiously viewed the participant through a concealed window, and re-entered the testing room once the participant had completed the task.

Within each condition of Experiments 1 and 2A various controls were put in place to ensure that there were no biases towards copying a particular model, or towards copying a particular strategy depending on the order in which it was presented. More specifically, in the inefficient strategy conditions of Experiment 1 the controls included: 1) counterbalancing the order (first or second) in which each model demonstrated the task, and 2) counterbalancing the identity of the model who presented the box to the participants. In the mixed strategy conditions of Experiments 1 and 2A the controls included: 1) systematically varying the identity of the model who played the role of the inefficient model and the efficient model, 2) counterbalancing the order in which the participants witnessed the efficient strategy (first or second in Experiment 1; first, second, third or fourth in Experiment 2A), and 3) ensuring that the box was presented to the participants equally often by an inefficient model and the efficient model. Preliminary analyses revealed that the participants' performance across experiments was not biased by a particular model, or by the order in which a strategy was presented. Similarly there was no influence of observer sex on task performance therefore these factors were excluded from the main analyses.

### Data analysis

The mean number of irrelevant actions performed in the both models inefficient conditions (n = 3) and the mixed strategy conditions (n = 4) of Experiment 1 were analyzed separately using two univariate ANOVAs with condition (one, two or no-models present) as a between-participants factor. Similarly, the data from Experiment 2A was analyzed using a univariate ANOVA with condition (model majority or strategy majority) as the between participants factor. The data from these 9 conditions were then collapsed into 3 groups (inefficient majority conditions 2∶0 or 3∶1, and strategy equal conditions 1∶1) and analyzed initially using a between participants ANOVA, before a series of post hoc Tukey tests (with a Bonferroni correction applied, adapted significance level .0167 for 3 comparisons) were conducted in order to explore whether there were differences between the three condition types. In order to explore whether the removal of the experimental context in Experiment 2B influenced the number of irrelevant actions performed we conducted two repeated measures ANOVAs with box (1 or 2) as the within-participants factor (one ANOVA each for the model majority and strategy majority conditions). The final analysis (the results of which are presented in Table 2) was a cross condition comparison of performance in each of the 11 conditions of Experiments 1 and 2. The data were initially subjected to a univarite ANOVA with condition as a between-participants factor before a series of Post Hoc Tukey LSD tests were conducted (with a Bonferroni correction applied, adapted significance level .0009 for 55 comparisons).

### Coding and Reliability

The performance of the participants was videotaped using a camera which was positioned unobtrusively outside of the observer's line of sight. The data from twenty individuals representing 17% of the overall sample was re-analyzed by a naïve coder. This revealed that there was a great deal of consistency between the raters with respect to the occurrence of bolt removals and irrelevant actions (Cohen's κ = 1.0 in both cases).

## Supporting Information

Table S1 **Column 1 lists the individual conditions of Experiments 1 and 2A.** Column 2 indicates whether or not the majority of models were inefficient. Column 3 indicates whether or not the majority of strategies were inefficient. Column 4 indicates whether or not there was an inefficient model present during testing. Column 5 indicates whether or not the observer viewed an efficient strategy. √ indicates the presence of a factor; X indicates the absence of a factor. Colum 6 shows whether or not (Yes/No) over-imitation was witnessed. Numbers in brackets indicate the number of models present during testing.
(DOC)

Table S2 **Columns 1 and 2 list the individual conditions and the experiment in which they took place.** Column 3 shows the mean number of irrelevant actions performed listed from highest to lowest. Columns 4–13 show the cross condition comparisons revealed by post hoc Tukey LSD (with Bonferroni correction applied, adapted significance level .0009) tests following a univariate ANOVA with condition as a between participants factor where * is significant. Numbers in brackets indicate the number of models present during testing.
(DOC)

Table S3 **Proportion of participants who performed: 1) all five irrelevant actions, 2) some of the bolt removals and irrelevant taps, 3) the bolt removals only and 4) no irrelevant actions in each condition of Experiments 1 and 2.** Numbers in brackets indicate the number of models present during testing.
(DOC)
