# Supplementary material for: Is the Cultural Transmission of Irrelevant Tool Actions in Adult Humans (Homo Sapiens) Best Explained as the Result of an Evolved Conformist Bias?
Source: PLoS One. 2012 Dec 12;7(12):e50863. doi: 10.1371/journal.pone.0050863 (PMC3520947; doi:10.1371/journal.pone.0050863)
Supplement: Table S1 — Column 1 lists the individual conditions of Experiments 1 and 2A. Column 2 indicates whether or not the majority of models were inefficient. Column 3 indicates whether or not the majority of strategies were inefficient. Column 4 indicates whether or not there was an inefficient model present during testing. Column 5 indicates whether or not the observer viewed an efficient strategy. √ indicates the presence of a factor; X indicates the absence of a factor. Colum 6 shows whether or not (Yes/No) over-imitation was witnessed. Numbers in brackets indicate the number of models present during testing. (DOC) [file pone.0050863.s001.doc]

| Condition | Inefficient model  majority | Inefficient strategy majority | Inefficient model(s) present at test | Efficient strategy demonstrated | Over-imitation witnessed |
| --- | --- | --- | --- | --- | --- |
| Experiment 1 |  |  |  |  |  |
| Inefficient majority (0) | √ | √ | X | X | Yes |
| Inefficient majority (1) | √ | √ | √ | X | Yes |
| Inefficient majority (2) | √ | √ | √ | X | Yes |
| Mixed strategy (0) | X | X | X | √ | No |
| Mixed strategy, inefficient (1) | X | X | √ | √ | No |
| Mixed strategy, efficient (1) | X | X | X | √ | No |
| Mixed strategy (2) | X | X | √ | √ | No |
| Experiment 2A |  |  |  |  |  |
| Model majority (4) | √ | √ | √ | √ | Yes |
| Strategy majority (2) | X | √ | √ | √ | Yes |

Table S1. Column 1 lists the individual conditions of Experiments 1 and 2A. Column 2 indicates whether or not the majority of models were inefficient. Column 3 indicates whether or not the majority of strategies were inefficient. Column 4 indicates whether or not there was an inefficient model present during testing. Column 5 indicates whether or not the observer viewed an efficient strategy. √ indicates the presence of a factor; X indicates the absence of a factor. Colum 6 shows whether or not (Yes/No) over-imitation was witnessed. Numbers in brackets indicate the number of models present during testing.
