# Supplementary material for: Is the Cultural Transmission of Irrelevant Tool Actions in Adult Humans (Homo Sapiens) Best Explained as the Result of an Evolved Conformist Bias?
Source: PLoS One. 2012 Dec 12;7(12):e50863. doi: 10.1371/journal.pone.0050863 (PMC3520947; doi:10.1371/journal.pone.0050863)
Supplement: Table S2 — Columns 1 and 2 list the individual conditions and the experiment in which they took place. Column 3 shows the mean number of irrelevant actions performed listed from highest to lowest. Columns 4–13 show the cross condition comparisons revealed by post hoc Tukey LSD (with Bonferroni correction applied, adapted significance level .0009) tests following a univariate ANOVA with condition as a between participants factor where * is significant. Numbers in brackets indicate the number of models present during testing. (DOC) [file pone.0050863.s002.doc]

| Condition | | Exp. | Mean | 2 | 3 | 4 | 5 | *6* | *7* | *8* | *9* | 10 | 11 |
| --- | --- | --- | --- | --- | --- | --- | --- | --- | --- | --- | --- | --- | --- |
| 1 | Inefficient majority (2) | 1 | 4.2 | n.s. | n.s. | n.s. | n.s. | ***** | ***** | ***** | ***** | ***** | ***** |
| 2 | Inefficient majority (1) | 1 | 4.1 | - | n.s. | n.s. | n.s. | n.s. | ***** | ***** | ***** | ***** | ***** |
| 3 | Four models (4) | 2A | 3.2 |  | - | n.s. | n.s. | n.s. | n.s. | ***** | ***** | ***** | ***** |
| 4 | Inefficient majority (0) | 1 | 2.8 |  |  | - | n.s. | n.s. | n.s. | n.s. | n.s. | ***** | ***** |
| 5 | Two models (2) | 2A | 2.4 |  |  |  | - | n.s. | n.s. | n.s. | n.s. | n.s. | ***** |
| 6 | Mixed strategy (0) | 1 | 1.7 |  |  |  |  | *-* | n.s. | n.s. | n.s. | n.s. | n.s. |
| 7 | Mixed strategy I stays (1) | 1 | 0.9 |  |  |  |  |  | *-* | n.s. | n.s. | n.s. | n.s. |
| 8 | Mixed strategy E stays (1) | 1 | 0.8 |  |  |  |  |  |  | *-* | n.s. | n.s. | n.s. |
| 9 | Mixed strategy (2) | 1 | 0.7 |  |  |  |  |  |  |  | - | n.s. | n.s. |
| 10 | Four models Box 2 | 2B | 0.5 |  |  |  |  |  |  |  |  | - | n.s. |
| 11 | Two models Box 2 | 2B | 0.1 |  |  |  |  |  |  |  |  |  | - |

Table S2. Columns 1 and 2 list the individual conditions and the experiment in which they took place. Column 3 shows the mean number of irrelevant actions performed listed from highest to lowest. Columns 4-13 show the cross condition comparisons revealed by post hoc Tukey LSD (with Bonferroni correction applied, adapted significance level .0009) tests following a univariate ANOVA with condition as a between participants factor where * is significant . Numbers in brackets indicate the number of models present during testing.
