# Supplementary material for: Is the Cultural Transmission of Irrelevant Tool Actions in Adult Humans (Homo Sapiens) Best Explained as the Result of an Evolved Conformist Bias?
Source: PLoS One. 2012 Dec 12;7(12):e50863. doi: 10.1371/journal.pone.0050863 (PMC3520947; doi:10.1371/journal.pone.0050863)
Supplement: Table S3 — Proportion of participants who performed: 1) all five irrelevant actions, 2) some of the bolt removals and irrelevant taps, 3) the bolt removals only and 4) no irrelevant actions in each condition of Experiments 1 and 2. Numbers in brackets indicate the number of models present during testing. (DOC) [file pone.0050863.s003.doc]

| Condition | All irrelevant actions | Partial bolts and taps | Bolts only | Relevant actions only |
| --- | --- | --- | --- | --- |
| Inefficient majority (0) | 0.42 | 0.16 | 0.16 | 0.25 |
| Inefficient majority (1) | 0.58 | 0.25 | 0.16 | 0 |
| Inefficient majority (2) | 0.67 | 0.25 | 0 | 0.08 |
| **Total** | **0.56** | **0.22** | **0.11** | **0.11** |
| Mixed strategy (0) | 0.25 | 0 | 0.25 | 0.50 |
| Mixed strategy (1-inefficient) | 0 | 0.16 | 0.16 | 0.67 |
| Mixed strategy (1-efficient) | 0.08 | 0.08 | 0.08 | 0.75 |
| Mixed strategy (2) | 0 | 0 | 0.33 | 0.67 |
| **Total** | **0.08** | **0.06** | **0.21** | **0.65** |
| Model Majority (4) | 0.40 | 0.27 | 0.13 | 0.20 |
| Strategy majority (2) | 0.44 | 0 | 0.12 | 0.44 |
| **Total** | **0.42** | **0.13** | **0.13** | **0.32** |
| Box 2 (Model Majority ) | 0.07 | 0 | 0.07 | 0.86 |
| Box 2 (Strategy Majority) | 0 | 0.06 | 0 | 0.94 |
| **Total** | **0.03** | **0.03** | **0.03** | **0.90** |

Table S3. Proportion of participants who performed: 1) all five irrelevant actions, 2) some of the bolt removals and irrelevant taps, 3) the bolt removals only and 4) no irrelevant actions in each condition of Experiments 1 and 2. Numbers in brackets indicate the number of models present during testing.
